# Physical Activity and Quality of Life of University Students, Their Parents, and Grandparents in Poland—Selected Determinants

**DOI:** 10.3390/ijerph18083871

**Published:** 2021-04-07

**Authors:** Katarzyna Kotarska, Maria Alicja Nowak, Leonard Nowak, Paweł Król, Artur Sochacki, Katarzyna Sygit, Marian Sygit

**Affiliations:** 1 Institute of Physical Culture Sciences, Faculty of Physical Culture and Health, University of Szczecin, 71-065 Szczecin, Poland; katarzyna.kotarska@usz.edu.pl (K.K.); maria.nowak@usz.edu.pl (M.A.N.); leonard.nowak@usz.edu.pl (L.N.); 2 Institute of Physical and Cultural Studies, Medical College of Rzeszów University, 35-959 Rzeszów, Poland; krolpawel1@poczta.onet.pl (P.K.); artisocha@wp.pl (A.S.); 3 Faculty of Health Sciences, Calisia University, 62-800 Kalisz, Poland; msygit@onet.pl

**Keywords:** lifestyle, training, exercises, intergenerational relations, academic youth, standardized questionnaire WHOQOL-BREF

## Abstract

Physical activity is one of the factors conditioning human health. Research shows a positive impact of regular physical activity on the quality of human life. The aim of the study was to determine the relationship between the physical activity of university students, their parents, and their grandparents, and the overall quality of their lives in individual domains (physical, mental, social, environmental), as well as the perceived state of health in relation to selected determinants. The research included 1001 participants, including 253 students related to physical culture and health promotion studying at the University of Szczecin (faculties: physical education, tourism and recreation, public health, sports diagnostics), and their 336 parents and 412 grandparents. Purposive sampling was used to outline the determinants of quality of life and family factors in physical activity. The diagnostic survey was carried out based on the standardized WHOQOL-BREF (World Health Organization Quality of Life) questionnaire. Statistically significant differences were shown in the studied generations regarding the assessment of quality of life and satisfaction with health in the physical, psychological, social, and environmental domains. The oldest generation gave the lowest assessment of quality of life and was the least satisfied with their health regarding particular domains. Female students were more satisfied with their health compared to grandmothers and grandfathers, whereas male students compared to mothers and fathers. Fathers achieved the highest scores in the psychological and social domains, but, in case of the latter, differences were found between mothers’ and fathers’ assessments. Intergenerational differences were found in quality of life and the assessment of health status. Current participation in broadly understood physical culture was often a result of positive attitudes towards physical education and doing sport in the past, which meant higher scores in the physical domain each time. The study demonstrated that taking up physical activity impacted the quality of life and assessment of health in the past and currently.

## 1. Introduction

The concept of quality of life is wide, and there is no single definition of quality of life in either social sciences or medical sciences. Quality of life is studied in relation to a particular area of life and the factors that affect it. Usually, factors such as health, life satisfaction, happiness, psychosocial adjustment, well-being, or physical activity are taken into account [1,2,3]. 

According to the WHO (World Health Organization) definition, quality of life (QOL) is “an individual’s perception of their position in life in the context of the culture and value systems in which they live and in relation to their goals, expectations, standards and concerns” [1,3,4]. In medicine, health-related quality of life (HRQOL), introduced by Schipper et al., is used more often [1,2,5]. It is based on the WHO definition of health. The WHO defines health as follows: “Health is a state of complete physical, mental and social well-being and not merely the absence of disease or infirmity” (the WHO definition of Health, Preamble to the Constitution of the World Health Organization as adopted by the International Health Conference) [1,2,3,4,5]. 

Over the years, however, there have been various changes to the term HRQQL. Currently, most definitions address five dimensions of health-related quality of life. These are: physical, psychological, social, and cognitive functioning, as well as a general sense of well-being [1,2,3,6,7,8,9]. The medical view of the quality of life emphasizes the great influence of such factors as sex, age, education, or the level of physical activity [1,2,3,7,10].

The factors that influence quality of life undoubtedly include physical activity, which, among others, includes various kinds of games, exercises, or amateur practices of various sports disciplines as a part of active leisure. Physical activities of this type are carried out for various purposes: for pleasure, leisure, and to maintain health, improve exercise capacity, or acquire special physical abilities and skills. Such healthy behaviors prevent the occurrence of various civilization diseases [7,8,9,11]. It should be emphasized that physical activity needs to accompany a person throughout their life, which is why it is so important to promote physical culture not only among children, but also among adolescents, because it provides the basis for the continuation of healthy behaviors in adulthood [4,5,6,12]. When examining the quality of life of healthy adults, it may be concluded that physical activity is not only strictly related to physical condition, but also to mental condition [7,8,13,14,15,16,17]. 

The assessment of quality of life depends not only on the state of physical and mental health, level of independence, social relations, and environmental factors, but also on the individual attitude of a given person. Although the abovementioned definition of health according to the WHO clearly refers to quality of life, research on this subject was initiated relatively recently; in the 1990s, systematic studies in this field started to be undertaken [4,5,6,18,19,20,21,22]. It is known from today that these studies should take into account, among others, the degree of satisfaction with health and family life, social relations, professional life, education, and specialization or universal standards affecting the quality of life in the local community, which the authors have pointed out in this study [16,17,18,23,24,25,26,27,28,29]. 

In recent years, there has been a lot of research on quality of life in the population of healthy and sick people, which analyzed various factors, such as lifestyle, forms of leisure, or the socioeconomic situation. Adults with frequent foot problems should be given special attention. Foot pain affects one’s overall health, and often causes a person to fall. Studies conducted by scientists in Spain confirm that this is one of the reasons for the reduction of daily physical activity [30].

Taking into account the existing knowledge on quality of life and physical activity, we tried to determine how these relationships work in relation to university students (faculties of physical education, sports diagnostics, public health, tourism, and recreation), their parents, and their grandparents. The aim of the study was to determine the relationship between the physical activity of university students, their parents, and their grandparents, as well as the overall quality of their lives in individual domains (physical, mental, social, environmental) and the perceived state of health in relation to selected determinants.

A hypothesis was formulated that there is a relationship between current physical activity and quality of life within the individual generations. It was also assumed that the age, gender, marital status, education level, and employment status of respondents may modify this relationship. 

## 2. Methodology

### Participants and Ethical Considerations

The research was carried out on 1001 participants, including 253 students related to physical culture and health promotion studying at the University of Szczecin (faculties: physical education, tourism and recreation, public health, sports diagnostics), and their 336 parents and 412 grandparents. Female students (171) were aged 18–34 years, with an average of 22.4 ± 3.44. The average age of the 82 male students, aged 19–28 years, was 21.5 ± 2.0. The mothers of the studied (205) persons were aged 35–68 years, the average being 47.7 ± 7.4. For the 131 fathers, in the 35–77 age range, the average age was 49.6 ± 8.5. The average age for the 262 grandmothers, 51–99 years old, was 73.6 ± 8.9, and for the 150 grandfathers, 55–97 years old, it was 73.8 ± 8.9. Persons over 74 constituted 14.6%, including 3.3% over 90. The majority of the respondents were women (63.8%), and their predominance was significant in every age group. The unmarried respondents constituted 48.8% (including those never married 26.5%, widowed 14.8%, and divorced 7.5%). The respondents (including students) were most likely to have secondary education (46.7%). Grandfathers and grandmothers mostly had primary education (60.4% and 51%). Mothers of the students were better educated than their fathers. Above-secondary education was reported by 45.6% of mothers and 32.8% of fathers, and below-secondary education was reported by 37.4% of fathers and 19.1% of mothers. Employment was reported by over 70% of the surveyed students and their parents (over 72%), and by about 10% of the grandparents. Respondents described their financial situation as very good (39.2%), moderate (46.4%), sufficient (10.7%), or bad (3.7%). The majority of the respondents lived in medium and large cities; only a minority lived in rural areas (21.5%).

The study protocol was approved by the appropriate ethics committee of the Local Medical Chamber in Szczecin, permit no 15/KB/V.2015, and conformed to the ethical guidelines of the 1975 Declaration of Helsinki. Written informed consent was obtained from each subject included in the study.

## 3. Data Collection

The diagnostic survey method was based on the standardized WHOQOL-BREF (World Health Organization Quality of Life) questionnaire. An abbreviated version, adapted to Polish language, culture, and psychometric conditions, contained 26 questions [30]. The first two questions, relating to the individual’s general perception of the quality of life and health, were analyzed separately. The remaining 24 questions concerned four domains of the perceived quality of life. The physical domain included an assessment of daily life activities, drug dependence and treatment, energy and fatigue, mobility, pain and discomfort, rest and sleep, and the ability to work. The psychological domain concerned body image, joy, sense of life, mood, negative and positive feelings, self-esteem, and concentration/memory/thinking/learning. The social domain concerned personal relationships, sexual activity, and social support. The environmental domain concerned financial resources, safety, home, physical environment, acquiring new information and skills, recreation and leisure, housing conditions, and access to medical care and transportation. The scores for the aforementioned domains were determined by calculating the arithmetic mean of their constituent items. The higher the score, the higher the quality of life; the maximum value was 120 points. Our research also included our survey technique to study the lifestyles of women and men, as well as an interview. Information obtained from the interviews conducted among the students was used to supplement and verify the results of the research. Our study used personal data on physical activity, age, gender, place of residence, education, employment, and financial situation. The focus was on establishing a relationship between the quality of life and currently undertaking physical activity over the previous week and in the past (participation in physical education classes or involvement in professional sport) of the students of the Faculty of Physical Culture and Health Promotion of the University of Szczecin, their parents, and their grandparents. The quality of life of respondents were also analyzed in relation to socio-demographic variables.

## 4. Data Analysis

The one-way analysis of variance (F-test) for independent groups was used (ANOVA), as well as Student’s *t*-test. The effect size in ANOVA was expressed using ω^2^. For the Student’s *t*-test, we used Hedges’ g—a measure of effect size. In qualitative analyses, the trait frequency and the chi-square test of independence were used, as well as Cramér’s V for the χ^2^ test. Differences were deemed statistically significant at *p* ≤ 0.05. Statistical calculations were made with Statistica 12 for Windows, Microsoft Excel 2007, and JASP 0.10.0.0 [31,32].

## 5. Results

**Figure 1:**
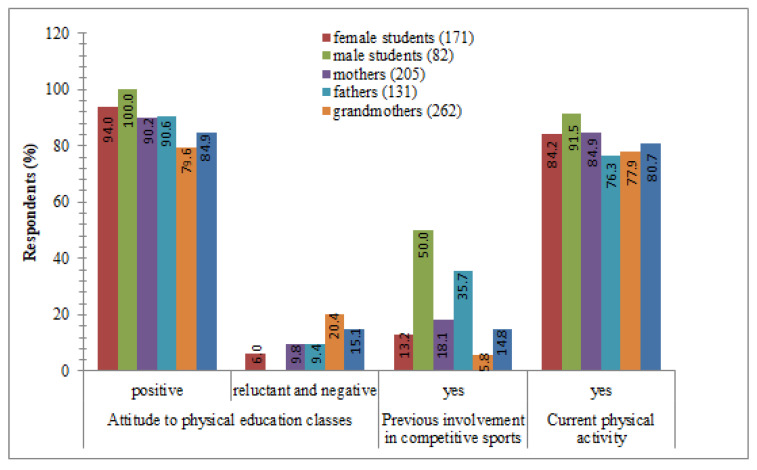
Physical activity characteristics of university students, their parents, and their grandparents (independence χ2 test and Cramér’s V).

The attitudes of the respondents towards compulsory participation in physical education classes, practicing professional sport in the past, and current recreational physical activity are presented in Figure 1.

The majority of the respondents had willingly participated in physical education classes in the past (88.1%). A reluctant and negative attitude towards those lessons was more often shown by the oldest generation (grandmothers 20.4%; grandparents 15.1%). Few respondents (2%) had been exempted from these classes. All of the university students willingly participated in compulsory physical education classes (94% of women and 100% of men). According to research, 18.4% of respondents declared involvement in competitive sport in the past, men more often than women (students 50%; fathers 35.7%; grandparents 14.8%) (*p* < 0.001 for the χ^2^ test, Cramér’s V = 0.3). As a result of the analysis, it was found that 81.7% of respondents declared current physical activity. The percentages of physically active female students and their mothers were similar. Grandmothers declared lower physical activity (77.9%). Male students were physically more active compared to fathers and grandparents (91.5% vs. 76.3% vs. 80.7%, respectively) (*p* < 0.05 for the χ^2^ test, Cramér’s V = 0.1). 

**Figure 2:**
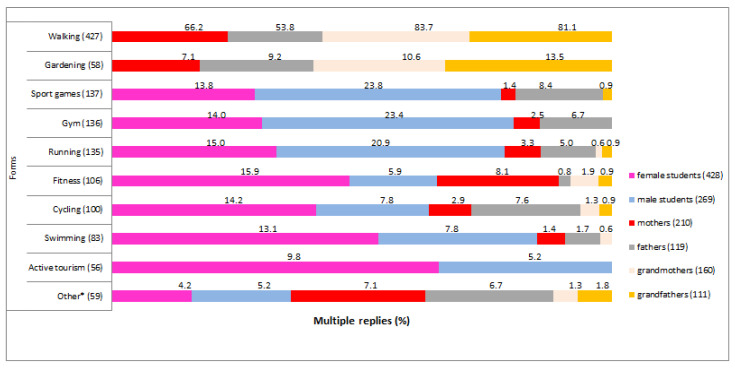
Physical activity patterns of students, their parents, and their grandparents (%). * Combat sports, dance, pole dance, horseback riding, tennis, table tennis, and badminton.

Respondents varied slightly in their preferences regarding the forms of physical activity (Figure 2). Students attended fitness classes, ran, cycled, went to the gym, played sports games, and swam (about 15% each). Students found sports games, gym exercises, and jogging more attractive than the older generations. Mothers (66.2%), fathers (53.8%), grandmothers (83.7%), and grandparents (81.1%) most often walked. This form of activity was not even mentioned by university students. Gardening was more significant for parents and grandparents, who often treated this activity as a form of recreation. Some mothers also attended fitness classes (8.1%), and some fathers played sports games (8.4%). 

The evaluation of quality of life, health satisfaction, and physical, psychological, social, and environmental domains in the studied generations revealed statistically significant differences (*p* < 0.001 for the F-test). In the physical domain (ω² = 0.18) and health satisfaction (ω² = 0.11), the effect size was high and close to high, respectively, and in the quality of life satisfaction and in the other domains, it was moderate or close to moderate (in the environmental domain) (Table 1). 

Statistically significant differences were found in satisfaction with life between female students and their grandparents, and between parents and grandparents of male students (*p* < 0.001 for the Student’s *t*-test). Grandmothers and grandparents had the lowest scores for quality of life and in the individual domains, as well as in satisfaction with health. Grandmothers were less satisfied with their health than grandfathers (*p* < 0.05 for the Student’s *t*-test). 

Female students were more satisfied with their health than grandmothers and grandparents, and male students were more satisfied with their health than mothers (*p* < 0.05 for the Student’s *t*-test) and fathers (*p* < 0.05 for the Student’s *t*-test). Female students were less satisfied with their health than male students (*p* < 0.05 for the Student’s *t*-test). In the psychological domain, we found differences between female students and fathers (*p* < 0.001 for the Student’s *t*-test), and between mothers and fathers (*p* < 0.05 for the Student’s *t*-test). In this domain, fathers achieved the highest scores, as well as in the social domain (*p* < 0.05 for the Student’s *t*-test). The moderate and high effect size (Hedges’ g = 0.5–1.0) confirmed the differences between students and grandparents in the assessment of satisfaction of life, satisfaction with health, and in individual domains (physical, psychological, social, and environmental). In the physical domain, the effect size was very high (g = 0.8–1.1). These differences, confirmed by the effect size, were also observed between the students’ parents and grandparents (g = 0.8–0.9). 

The reported quality of life differed between those who had participated in physical education, professional sport, and recreational activities in the past (Table 2).

Those willingly participating in physical education classes had a higher quality of life rating (*p* < 0.01) and scored better in the physical (*p* < 0.01) and social domain (*p* < 0.01). In a comparison of those involved in competitive sport in the past and those who were not, the former reported higher satisfaction of life (*p* < 0.001), health satisfaction (*p* < 0.001), and in physical (*p* < 0.001), psychological (*p* < 0.001), and social domains (*p* < 0.01). Those currently engaged in physical recreation were also more satisfied with their health (*p* < 0.001), performing better in the physical (*p* < 0.001), psychological (*p* < 0.001), and environmental domains (*p* < 0.05) than those who were not currently physically active. Their effect size was low (g = 0.2–0.3). 

General differences in quality of life were found in relation to the marital status of the respondents (*p* < 0.001 for F-test) (Table 3).

The lowest mean scores for quality of life, satisfaction with health, and its components were characteristic of widowed and divorced persons. Unmarried persons, as well as those married and in cohabitation, rated their quality of life higher than widowed people. Divorced persons also had a higher quality of life than widowers. The effect size on the perception of quality of life among unmarried persons and those living in cohabitation (g = 0.8) compared to widows/widowers was very high, as was a higher satisfaction with health among unmarried persons in comparison with widowed persons (g = 0.8). In the physical and social domains, widowed persons had lower scores than unmarried and married persons and those living in a cohabitation (g = 0.6–1.1). In the psychological and environmental domains, widowed persons were rated the lowest in comparison with the other groups (moderate effect size).

There were statistically significant differences in quality of life among the studied students, their parents, and their grandparents, depending on the level of education (Table 4).

The overall variation in satisfaction with life, health, and in the physical domain (F-test) was confirmed by the moderate effect size (ω² = 0.06). Those with secondary education, especially post-secondary education, had higher quality of life scores, were more satisfied with their health, and achieved higher results in the remaining domains (Hedges’ g within the range −0.3 to −0.6).

Employed persons (*n* = 440) had a higher quality of life and health satisfaction and better results in all domains in comparison to unemployed persons (*p* < 0.001 for F-test, effect size high and moderate, as shown by ω² = 0.15 to 0.06) (Table 5).

In each case, statistically significant differences were found in quality of life and its components, depending on the employment status (*p* < 0.001 for the *t*-test). These differences were confirmed by the moderate and above-moderate effect size (g = 0.5 to 0.7), and were very high in the physical domain (g = 0.8).

## 6. Discussion

Scientific literature provides unequivocal evidence that physical fitness resulting from physical activity is a significant predictor of health [11,12,13,14]. Good health and fitness are important determinants of professional and social competences, as well as better quality of life. In Poland, a survey conducted at the beginning of 2019 on a representative random sample of 1858 individuals aged 15 and over revealed that 64% declared partaking in physical activity at least once a month, and 19% exercised systematically, i.e., five times a week. In comparison with the survey of 1800 Poles in the previous year, the 2019 results showed a two percentage point increase in people spending their time actively at least once a month (walking, cycling, running, gym exercises, fitness classes). However, this still means that more than one third of Poles did not engage in any physical activity, even once a month. Most physically active were young people aged 15 to 24 (80%), students (90%), those with university degrees (78%), and those with monthly incomes exceeding PLN 5000 (83%), i.e., 20% above the average wage in Poland [15].

Dependencies between physical activity and quality of life are not always unequivocal. Scientific research has repeatedly shown the positive impact of physical activity on quality of life, such as in university students. The increased physical activity resulted in an improved quality of life [17]. At the same time, some studies do not confirm any relationship between a very high level of physical activity for students and their quality of life and self-esteem, with some papers highlighting the combined influence of other factors, such as gender, age, and marital status [25,26]. A greater effect of frequent exercise on quality of life has been observed in adult pre-diabetics and women with depressive symptoms [27]. Exercise can also improve the quality of life of patients with Alzheimer’s disease and following transplants of the liver, kidney, and other organs [28].

A study carried out in the years 2014–2015 among 4460 residents of Wrocław, aged 18–64, determined the relationship between physical activity and quality of life using the IPAQ (short version) and WHOQOL-BREF [29]. The authors found that the highest overall quality of life, health perception, and quality of life in the physical, psychological, social, and environmental domains were reported by respondents of both genders whose activity levels were defined as high (at least 1500 MET/min/week). In the case of low physical activity, the correlations were lower.

The results partly corroborate the hypothesis about the relationships between the levels of physical activity among students, their parents, and their grandparents, the overall quality of their lives in individual domains (physical, psychological, environmental), and their perceived health status. The quality of life of respondents was dependent upon, among other things, their current physical activity levels. This was a result of positive attitudes towards physical education lessons (in the past) and competitive sports (students at present, parents, and grandparents in the past). Those currently engaged in physical recreation were also more satisfied with their health, performing better in the physical, psychological, and environmental domains than those who were not currently physically active. The moderate effect size confirms these differences, especially concerning the physical and psychological domains. Individuals declaring some physical activity performed significantly better in these domains. There was no such dependence in the social domain. 

Research carried out in many countries highlights the importance of participating in physical education classes [31,32,33]. Our research confirms these results. Those currently participating in physical recreation (81.7%) had positive attitudes towards physical education lessons (88.1%). They showed higher physical activity compared to all Poles (just over 70%) [17,34]. The positive attitudes of parents and grandparents likely encouraged interest in physical activity and sports activities among the surveyed students. These hypothetical relationships will be confirmed by further research.

Shaping positive attitudes towards exercise among teenagers is very important (especially among less physically active girls and women), because positive supportive attitudes towards physical activity result in sustained physical activity in later life and improved quality of life [4,35,36,37,38]. In one study, fathers and mothers had a similar effect on the level of physical activity of their children, regardless of their gender [39]. Nevertheless, girls do still exhibit lower levels of physical activity [40].

The general results of research somewhat confirm the MultiSport Index (2018), showing that the group of active people was mainly young people, educated people, people from large cities, and students. The highest inactivity rate was among pensioners (59%) and adults over 60 years of age (56%). Age is the factor that most strongly determines physical activity in Poles. The older a person is, the more likely he or she is to be less active [2,41]. The types of physical activity also differed between the sexes, regardless of the generation, which confirms the results of a previous study [37].

Physical activity among the elderly mainly consisted of walking, while sports games, jogging, and the gym were most popular among students. Cycling was declared by 7.7% of the respondents, which is probably connected with their place of residence (almost 80% of the respondents lived in cities) and the low availability of bicycle paths in Poland. During the research, it was found that, in the last six months, 29% of the respondents cycled (Multisport Index 2018). This shows that the promotion of cycling as a form of transport in Poland could increase the overall physical activity of society [42].

Studies on the relationship between physical activity and quality of life emphasize the importance of various modifying factors [43]. Kupcewicz et al. found a relationship between physical activity and quality of life of people over 50, modified by their sense of agency [44]. Boerma et al. demonstrated a relationship between recommended levels of moderate or intensive physical activity and HRQOL (175,850 Americans) and the number of sick leave days used. The percentage of adults reporting at least 14 sick leave days (regardless of age, gender, or ethnic group) was significantly lower among those who achieved the recommended level of physical activity. Achieving the recommended levels of exercise in leisure time in the French population (2333 men and 3321 women) was associated with a higher health-related quality of life. Increased intensity of exercise improved the quality of life of the respondents [10,45]. A greater effect related to the improvement of quality of life was achieved among older people exercising relatively frequently (three times a week) [46,47].

In this study, it was observed that physical activity and quality of life were also modified by age, gender, marital status, education level, and employment status. University students in study exhibited a higher level of physical activity than parents and grandparents. Women were less physically active than men, except for fathers of students, who were currently the least active among men (76.3%), and their quality of life was also lower than that of students’ mothers (84.9%). Lower physical activity of students’ fathers may have resulted from a high employment rate (over 72%), but, at the same time, 35.7% of fathers declared their previous involvement in competitive sports. Their low participation in physical recreation requires further research. 

Many studies confirm the negative impact of age on people’s quality of life, but the social, intellectual, and physical activity of older adults, while engaging in activities for the benefit of the local environment, brings a positive increase in quality of life, especially in the physical and social fields [17,29,30,48].

The greatest differences in the quality of life were found between female and male students and their grandmothers and grandfathers, and between mothers and fathers and grandmothers and grandfathers of students. Grandmothers and grandfathers had the lowest satisfaction with life and health, and in individual domains. Grandmothers were less satisfied with their health than grandfathers—otherwise, there were no differences between them in the physical, social, and environmental domains. Among men (fathers and grandfathers), the quality of life was significantly higher than among women (mothers and grandmothers). In the group of students, the impact of gender on quality of life was not so pronounced; it became more apparent in later stages of life.

In a survey conducted in 59 countries, data on the health status of people aged 18 and over (143,363 men and 115,321 women) were presented. Differences between men and women in assessing their health were found in all regions of the world. The authors suggest that combined biological and social factors (gender inequality) are the main causes of lower health assessment by women [45].

Differences between unmarried, married, and cohabiting persons were shown in comparison to widowed and divorced persons, who had the lowest quality of life, health satisfaction, and satisfaction in the physical, social, psychological, and environmental domains. Divorced persons also had a higher quality of life than widowers. These relationships were confirmed by the high and moderate strength of the effect. Villas-Boas, et al. proved that, among the predictors of quality of life for people of different ages, marital status (for middle-aged people) and income (for youth and older people) were important, but the most important was social support (important for all generations) [1].

The level of education in this study directly impacted the reported quality of life of the studied groups of students, their parents, and their grandparents. People with secondary education, especially post-secondary education, had a higher quality of life rating, were more satisfied with their health, and, in the remaining areas, achieved higher scores (moderate effect size).

Education also mitigates the differences in quality of life resulting from marital status [49]. A higher level of education is a positive determinant of quality of life.

Those employed had a higher quality of life, health satisfaction, and better scores in all areas compared to the unemployed (high and moderate effect size). In each case, statistically significant differences were found in the quality of life and its components, depending on the employment status (moderate and high effect size).

Among women, higher satisfaction with health was declared by women who were better educated, employed, or married. According to research conducted by the Ministry of Labour and Social Policy (2008), health status varies significantly depending on the employment status. The highest declared health condition was reported by employees at the age around retirement, 70% of whom described it as good or very good. According to 7% of Poles and 14% of Europeans, poor health may not only make it difficult to work, but also make it impossible to take up or continue a job. Deactivation changes their situation, deprives people of professional contacts, and thus significantly forces them to change their behavior and habits, which in turn reduces their satisfaction with life in retirement [50,51,52].

The results of the study showed that the grandparents of the respondents often have limitations in the field of physical activity that affect their quality of life. As shown in studies by E. Navarro-Flores et al., aging and chronic diseases, such as hyperglycemic disease, as well as musculoskeletal and cardiac processes, may cause the weakness syndrome and, consequently, become degenerative and show certain changes that may affect mental and general health. For example, aging and weakness may affect one’s walking speed and increase the risk of falling due to balance changes. Moreover, the presence of weakness symptoms influences health-related quality of life (HQoL) in this population group [53].

The results of this study, which were discussed on the basis of studies carried out by other authors, clearly showed statistically significant differences in physical activity and quality of life of academic youth, their parents, and their grandparents. The essence of our study is the inclusion of the parents and grandparents of the respondents, which gives a broader picture in terms of the researched subject matter.

The continuation of this study shall include the selection of the sample consisting of students in other fields of study; it will be carried out as part of the research project at selected universities throughout Poland.

## 7. Conclusions

Statistically significant differences were found in physical activity and quality of life of students, their parents, and grandparents. People who were currently physically active were more satisfied with their health, and achieved better results in the physical, psychological, and environmental domains. There were no such differences in the social domain.

The quality of life of students, their parents, and their grandparents was dependent on age, gender, marital status, level of education, and employment status. Higher quality of life was characteristic of younger, better educated, married or unmarried people, remaining in cohabitation, and being employed. Widows and widowers had the lowest overall quality of life in particular domains, and were the least satisfied with their health.

To improve quality of life in a society, it is necessary to promote various forms of physical activity aimed at meeting the needs of each generation, including gender, age, marital status, level of education, and employment status. Therefore, studies in physical culture and health promotion should educate health and physical activity leaders to be followed by people of all ages.

Practical implications:

Due to the need to increase physical activity of people of different ages (with particular emphasis on academic youth), which has been shown in the study to have a significant impact on quality of life, measures should be taken to focus on health education of the society, which can effectively eliminate the problem of incorrect health behaviors and encourage children, adolescents, adults and the elderly to engage in regular physical activity.

In this study, differences (confirmed by a moderate and high effect size) were found in the quality of life between university students and their grandparents. The most important determinants of the quality of life were marital status, employment status, and level of education. Those who were currently physically active rated the quality of their lives higher than inactive persons, but the effect size was low. We used a questionnaire concerning the attitudes of respondents towards participation in physical education classes, practicing professional sport, and current recreational physical activity. The use of a standardized questionnaire in future studies would enable broader comparisons to be made between the quality of life of physically active and passive people. Moreover, extending the list of determinants of quality of life to include the frequency of family contact and financial situation would help to more precisely establish the significance of physical activity and the life choices made by respondents concerning their quality of life.

## Figures and Tables

**Table 1:** Total quality of life in individual domains (physical, psychological, social, environmental) and satisfaction with health among university students, their parents, and their grandparents (F-test, ω², Student’s *t*-test, Hedges’ g).

Specification	Groups *	Value of *p* for Student’s *t*-Test					Hedges’ g					Means
		MS	M	F	GM	GF	MS	M	F	GM	GF	
Quality of life F = 16.87 *p* < 0.001 ω² = 0.07	FS	0.578	0.949	0.751	0.000	0.000	−0.1	0.1	0.1	0.6	0.5	4.03
	MS		0.516	0.438	0.000	0.000		0.1	0.1	0.7	0.7	4.09
	M			0.775	0.000	0.000			0.1	0.6	0.6	4.02
	F				0.000	0.000				0.5	0.5	4.00
	GM					0.859					−0.1	3.57
	GF											3.59
Satisfaction with health F = 26.42 *p* < 0.001 ω² = 0.11	FS	0.031	0.521	0.361	0.000	0.000	−0.3	0.1	0.1	0.8	0.5	3.83
	MS		0.011	0.012	0.000	0.000		0.3	0.4	1.0	0.8	4.04
	M			0.723	0.000	0.000			0.1	0.7	0.5	3.78
	F				0.000	0.000				0.7	0.4	3.75
	GM					0.034					−0.2	3.19
	GF											3.37
Physical domain F = 46.21 *p* < 0.001 ω² = 0.18	FS	0.598	0.459	0.684	0.000	0.000	0.1	01	0.1	1.1	0.8	28.58
	MS		0.923	0.921	0.000	0.000		0.1	−0.1	0.9	0.9	28.33
	M			0.823	0.000	0.000			−0.1	0.9	0.8	28.28
	F				0.000	0.000				0.9	0.8	28.39
	GM					0.294					−0.1	23.89
	GF											24.41
Psychological domain F = 15.94 *p* < 0.001 ω² = 0.07	FS	0.064	0.153	0.000	0.000	0.055	−0.2	−0.1	−0.4	0.4	0.2	21.53
	MS		0.466	0.282	0.000	0.001		0.1	−0.2	0.7	0.5	22.46
	M			0.032	0.000	0.001			−0.2	0.6	0.4	22.10
	F				0.000	0.000				0.8	0.6	23.05
	GM					0.057					−0.2	19.88
	GF											20.67
Social domain F = 20.93 *p*< 0.001 ω² = 0.09	FS	0.775	0.409	0.118	0.000	0.000	0.1	0.1	−0.2	0.7	0.4	11.61
	MS		0.713	0.095	0.000	0.000		0.1	−0.2	0.6	0.5	11.51
	M			0.013	0.000	0.000			−0.3	0.6	0.4	11.40
	F				0.000	0.000				0.9	0.7	12.04
	GM					0.150					−0.1	9.97
	GF											10.33
Environmental domain F = 9.34 *p* < 0.001 ω² = 0.04	FS	0.423	0.371	0.599	0.000	0.000	0.1	0.1	0.1	0.5	0.4	30.43
	MS		0.955	0.809	0.000	0.006		−0.1	−0.1	0.4	0.4	29.99
	M			0.813	0.000	0.000			−0.1	0.4	0.4	30.02
	F				0.000	0.002				0.4	0.4	30.15
	GM					0.577					−0.1	28.12
	GF											28.38

^*^ The following abbreviations were used: female students—FS, male students—MS, mothers—M, fathers—F, grandmothers—GM, grandfathers—GF.

**Table 2:** Physical activity of respondents in the past and present and their quality of life (F-test, ω², Student’s *t*-test, Hedges’ g).

Specification	Physical Activity	Value of *p* for Student’s *t*-Test	Hedges’ g	Means
Participation in physical education classes				
Satisfaction with life F = 6.98, *p* < 0.01 ω² = 0.01	Willing	0.008	0.3	3.87
	Reluctant and negative			3.65
Physical domain F = 7.72, *p* < 0.001 ω² = 0.01	Willing	0.005	0.3	26.79
	Reluctant and negative			25.34
Social domain F = 6.84, *p* < 0.01 ω² = 0.01	Willing	0.009	0.3	11.07
	Reluctant and negative			10.37
Practicing competitive sport				
Satisfaction with life F = 10.12, *p* < 0.001 ω² = 0.01	Yes	0.001	0.3	4.01
	No			3.80
Satisfaction with health F = 12.01, *p* < 0.001 ω² = 0.01	Yes	0.000	0.3	3.78
	No			3.54
Physical domain F = 12.16, *p* < 0.001 ω² = 0.01	Yes	0.000	0.3	27.74
	No			26.35
Psychological domain F = 13.26, *p* < 0.001 ω² = 0.01	Yes	0.000	0.3	22.33
	No			21.11
Social domain F = 6.56, *p* < 0.01 ω² = 0.01	Yes	0.010	0.2	11.39
	No			10.87
Current participation in physical recreation				
Satisfaction with health F = 10.48, *p* < 0.001 ω² = 0.01	Yes	0.001	0.3	3.63
	No			3.40
Physical domain F = 15.99, *p* < 0.001 ω² = 0.01	Yes	0.000	0.3	26.91
	No			25.32
Psychological domain F = 12.65, *p* < 0.001 ω² = 0.01	Yes	0.000	0.3	21.58
	No			20.40
Environmental domain F = 5.3, *p* < 0.05 ω² = 0.01	Yes	0.021	0.2	29.52
	No			28.66

**Table 3:** Marital status vs. quality of life reported by students, their parents, and their grandparents (F-test, ω², *t*-test, Hedges’ g).

Specification	Marital Status	Value of *p* for Student’s *t*-Test				Hedge’s g				Means
		Married	Divorced	Widowers	Cohabitation	Married	Divorced	Widowers	Cohabitation	
Quality of life F = 16.43 *p* < 0.001 ω² = 0.06	Unmarried	0.419	0.023	0.000	0.962	0.1	0.3	0.8	0.1	3.97
	Married		0.077	0.000	0.792		0.2	0.7	−0.1	3.92
	Divorced			0.001	0.197			0.5	−0.3	3.75
	Widowed				0.000				−0.8	3.39
	Cohabitation									3.96
Satisfaction with health F = 14.34 *p* < 0.001 ω² = 0.06	Unmarried	0.023	0.000	0.000	0.216	0.2	0.5	0.8	0.3	3.78
	Married		0.025	0.000	0.781		0.3	0.5	0.1	3.64
	Divorced			0.057	0.280			0.3	−0.2	3.40
	Widowed				0.017				−0.5	3.17
	Cohabitation									3.59
Physical domain F = 23.36 *p* < 0.001 ω² = 0.08	Unmarried	0.186	0.015	0.000	0.500	0.1	0.3	1.0	0.1	27.62
	Married		0.148	0.000	0.919		0.2	0.8	0.1	27.14
	Divorced			0.000	0.425			0.6	−0.2	26.25
	Widowed				0.000				−0.8	23.26
	Cohabitation									27.04
Psychological domain F = 14.78 *p* < 0.001 ω² = 0.06	Unmarried	0.015	0.576	0.000	0.626	−0.2	0.1	0.5	0.1	21.36
	Married		0.033	0.000	0.134		0.3	0.7	0.3	22.08
	Divorced			0.002	0.923			0.4	0.1	21.05
	Widowed				0.041				−0.4	19.24
	Cohabitation									20.96
Social domain F = 32.40 *p* < 0.001 ω² = 0.01	Unmarried	0.053	0.002	0.000	0.851	−0.1	0.4	0.8	0.1	11.21
	Married		0.000	0.000	0.311		0.6	1.1	0.2	11.56
	Divorced			0.001	0.089			0.4	−0.4	10.19
	Widowed				0.000				−0.9	9.14
	Cohabitation									11.11
Environmental domain F = 11.54 *p* < 0.001 ω² = 0.04	Unmarried	0.528	0.070	0.000	0.795	−0.1	0.2	0.6	−0.1	29.70
	Married		0.026	0.000	0.992		0.3	0.6	−0.1	29.92
	Divorced			0.026	0.228			0.3	−0.3	28.64
	Widowed				0.002				−0.6	27.18
	Cohabitation									29.93

**Table 4:** Education and quality of life of students, their parents, and their grandparents (F-test, ω², *t*-test, Hedges’ g).

Specification	Level of Education	Value of *p* for Student’s *t*-Test		Hedges’ g		Means
		Secondary	Post-Secondary	Secondary	Post-Secondary	
Quality of life F = 24.33 *p* < 0.001 ω² = 0.06	pre-secondary	0.000	0.000	−0.4	−0.6	3.61
	secondary		0.003		−0.2	3.89
	post-secondary					4.08
Satisfaction with health F = 21.39 *p* < 0.001 ω² = 0.06	pre-secondary	0.000	0.000	−0.4	−0.5	3.33
	secondary		0.214		−0.1	3.68
	post-secondary					3.76
Physical domain F = 28.21 *p* < 0.001 ω² = 0.06	pre-secondary	0.000	0.000	−0.5	−0.6	24.96
	secondary		0.093		−0.1	27.16
	post-secondary					27.81
Psychological domain F = 6.83 *p* < 0.001 ω² = 0.01	pre-secondary	0.273	0.000	−0.1	−0.3	20.93
	secondary		0.004		−0.2	21.25
	post-secondary					22.22
Social domain F = 14.71 *p* < 0.001 ω² = 0.01	pre-secondary	0.000	0.000	−0.3	−0.4	10.38
	secondary		0.281		−0.1	11.21
	post-secondary					11.42
Environmental domain F = 12.86 *p* < 0.001 ω² = 0.01	pre-secondary	0.007	0.000	−0.2	−0.4	28.49
	secondary		0.002		−0.3	29.38
	post-secondary					30.51

**Table 5:** Employment and quality of life for students, their parents, and their grandparents (F-test, ω², *t*-test, Hedges’ g).

Specification	Employment	Value of *p* for Student’s *t*-Test	Hedges’ g	Means
Quality of life F = 88.27 *p* < 0.001 ω² = 0.08	Yes	0.000	0.6	4.10
	No			3.64
Satisfaction with health F = 111.57 *p* < 0.001 ω² = 0.10	Yes	0.000	0.7	3.89
	No			3.34
Physical domain F = 170.08 *p* < 0.001 ω² = 0.15	Yes	0.000	0.8	28.75
	No			24.95
Psychological domain F = 67.47 *p* < 0.001 ω² = 0.06	Yes	0.000	0.5	22.51
	No			20.43
Social domain F = 82.16 *p* < 0.001 ω² = 0.08	Yes	0.000	0.6	11.78
	No			10.38
Environmental domain F = 55.03 *p* < 0.001 ω² = 0.06	Yes	0.000	0.5	30.53
	No			28.41

## Data Availability

Data are not publicly available and data sharing is not applicable to this article.

## References

[1] Villas-Boas S., Oliveira A.L., Natália Ramos N. (2019). Predictors of Quality of Life in Different Age Groups across Adulthood. J. Intergenerational Relatsh. (JIG).

[2] Nowak P.F., Bożek A., Blukacz M. (2019). Physical Activity, Sedentary Behavior, and Quality of Life among University Students. Biomed Res. Int..

[3] Núñez-Rocha G.M., López-Botello C.K., Salinas-Martínez A.M., Arroyo-Acevedo H.V., Martínez-Villarreal R.T., Ávila-Ortiz M.N. (2020). Lifestyle, Quality of Life, and Health Promotion Needs in Mexican University Students: Important Differences by Sex and Academic Discipline. Int. J. Environ. Res. Public Health.

[4] Tanabe T., Snyder A.R., Bay R.C., McLeod T.C.V. (2010). Representative values of health-related quality of life among female and male adolescent athletes and the impact of gender. Athl. Train. Sports Health Care.

[5] Snedden T.R., Scerpella J., Kliethermes S.A., Norman R.S., Blyholder L., Sanfilippo J., McGuine T.A., Heiderscheit B. (2019). Sport and Physical Activity Level Impacts Health-Related Quality of Life Among Collegiate Students. Am. J. Health Promot..

[6] Solis A.C., Lotufo-Neto F. (2019). Predictors of quality of life in Brazilian medical students: A systematic review and meta-analysis. Rev. Bras. Psiquiatr..

[7] Medrano-Ureña M.D., Ortega-Ruiz R., Benítez-Sillero J.D. (2020). Physical Fitness, Exercise Self-Efficacy, and Quality of Life in Adulthood: A Systematic Review. Int. J. Environ. Res. Public Health.

[8] Miko H.C., Zillmann N., Ring-Dimitriou S., Dorner T.E., Titze S., Bauer R. (2020). Effects of Physical Activity on Health. Gesundheitswesen.

[9] Ding D., Ramirez Varela A., Bauman A.E., Ekelund U., Lee I.M., Heath G., Katzmarzyk P.T., Reis R., Pratt M. (2020). Towards better evidence-informed global action: Lessons learnt from the Lancet series and recent developments in physical activity and public health. Br. J. Sports Med..

[10] Sevil J., Práxedes A., Abarca-Sos A., Del Villar F., García-González L. (2016). Levels of physical activity, motivation and barriers to participation in university students. J. Sports Med. Phys. Fit..

[11] Brown D.W., Balluz L.S., Heath G.W., Moriarty D.G., Ford E.S., Giles W.H., Mokdad A.H. (2003). Associations between recommended levels of physical activity and health-related quality of life. Findings from the 2001 Behavioral Risk Factor Surveillance System (BRFSS) survey. Prev. Med..

[12] Tittlbach S.A., Jekauc D., Schmidt S.C., Woll A., Bös K. (2017). The relationship between physical activity, fitness, physical complaints and BMI in German adults–results of a longitudinal study. Eur. J. Sport Sci..

[13] Bös K., Tittlbach S., Woll A., Suni J., Oja P. (2012). FinGer–Physicalactivity, fitness and health—An international longitudinal study in Bad Schönborn and Tampere. Int. Sports Stud..

[14] Rożek-Piechura K., Ignasiak Z., Sławińska T., Piechura J., Ignasiak T. (2014). Respiratory function, physical activity and body composition in adult rural population. Ann. Agric. Environ. Med..

[15] (2019). Physical activity of the society 2019. Physical and sporting activity of Poles. MultiSport Index.

[16] Koistinen S., Olai L., Ståhlnacke K., Fält A., Ehrenberg A. (2019). Oral health-related quality of life and associated factors among older people in short-term care. Int. J. Dent. Hyg..

[17] Çiçek G. (2018). Quality of Life and Physical Activity among University Students. Univers. J. Educ. Res..

[18] Allender S., Hutchinson L., Foster C. (2008). Life-change events and participation in physical activity: A systematic review. Health Promot. Int..

[19] Wang L., Qi J. (2016). Association between Family Structure and Physical Activity of Chinese Adolescents. BioMed Res. Int..

[20] Levasseur M., Desrosiers J., St-Cyr Tribble D. (2018). Do quality of life, participation and environment of older adults differ according to level of activity?. Health Qual. Life Out..

[21] Kruk J. (2014). Health and Economic Costs of Physical Inactivity. Asian Pac. J. Cancer Prev..

[22] Ribeiro O., Teixeira L., Araújo L., Rodríguez-Blázquez C., Calderón-Larrañaga A., Forjaz M.J. (2020). Anxiety, Depression and Quality of Life in Older Adults: Trajectories of Influence across Age. Int. J. Environ. Res. Public Health.

[23] Keating X.D., Guan J., Piñero J.C., Bridges D.M. (2005). A meta-analysis of college students’ physical activity behaviors. J. Am. Coll. Health.

[24] Włodarczyk K. (2015). Quality of life perceived by Poles in the 21st century. Konsumpcja Rozw..

[25] Gruszczyńska M., Skorupa P. (2018). Physical activity, quality of life and self-esteem. Probl. Hig. Epidemiol..

[26] An H.Y., Chen W., Wang C.W., Yang H.F., Huang W.T., Fan S.Y. (2020). The Relationships between Physical Activity and Life Satisfaction and Happiness among Young, Middle-Aged, and Older Adults. Int. J. Environ. Res. Public Health.

[27] Xu H., Tang L., Hu Z., Gao F., Yang Y., Qin L., Luo B.-A. (2018). Association between physical activity and health-related quality of life in elderly individuals with pre-diabetes in rural Hunan Province. China: A cross-sectional stud. BMJ Open.

[28] Kotarska K., Wunsch E., Raszeja-Wyszomirska J., Kempińska-Podhorodecka A., Wójcicki M., Milkiewicz P. (2015). Leisure time physical activity and health-related behaviours after liver transplantation: A prospective, single centre study. Gastroenterol. Rev..

[29] Puciato D., Rozpara M., Borysiuk Z. (2018). Physical Activity as a Determinant of Quality of Life in Working-Age People in Wrocław, Poland. Int. J. Environ. Res. Public Health.

[30] Navarro-Flores E., Losa-Iglesias M.E., Becerro-de-Bengoa-Vallejo R., López-López D., Rodríguez-Sanz D., Palomo-López P., Calvo-Lobo C. (2018). Translation and Test—Retest of the Spanish Podiatry Health Questionnaire (PHQ-S). Int. J. Environ. Res. Public Health.

[31] Hinkle D.E., Wiersma W., Jurs S.G. (2003). Applied Statistics for the Behavioral Sciences.

[32] R Core Team (2017). R: A Language and Environment for Statistical computing.

[33] Castro L., Svastisalee C., Mendes R., Fontaine O., Breda J. (2019). School-based physical activity and good practices in Europe. Health Probl. Civiliz..

[34] Word Health Organization (2010). Global Recommendations on Physical Activity for Health.

[35] Barengo N.C., Nissinen A., Tuomilehto J., Pekkarinen H. (2002). Twenty-five-year trends in physical activity of 30- to 59-year-old populations in eastern Finland. Med. Sci. Sports Exer..

[36] Beenackers M.A., Kamphuis C.B., Giskes K., Brug J., Kunst A.E., Burdorf A., van Lenthe F.J. (2012). Socioeconomic inequalities in occupational, leisure-time, and transport related physical activity among European adults: A systematic review. Int. J. Behav. Nutr. Phy. Act..

[37] Nowak M.A., Kotarska K., Nowak L. (2019). Physical Activity, Health and Physical Fitness of Students, Their Parents and Grandparents. Coll. Antropol..

[38] Al-Naggar R.A., Osman M.T., Musa R. (2003). Quality of Life among University Students in a Single Malaysian Institute. Pensee J..

[39] Maia J., Gomes T.N., Trégouët D.A., Katzmarzyk P.T. (2014). Familial Resemblance of Physical Activity Levels in The Portuguese Population. J. Sci. Med. Sport.

[40] Ajman H., Ukić M., Madić D. (2019). The relationship between family socio-economic status, family social support and adolescent physical activity. Health Probl. Civiliz..

[41] Central Statistical Office (CSO) Participation in Sports and Physical Recreation in 2016–2017. Statistical Information and Elaborations.

[42] Hosforda K., Fuller D., Lear A., Teschke K., Gauvin L., Brauer M. (2018). Evaluation of the impact of a public bicycle share program on population bicycling in Vancouver, BC. Prev. Med. Rep..

[43] Gill D.L., Hammond C.C., Reifsteck E.J., Jehu C.M., Williams R.A., Adams M.A., Lange E.H., Becofsky K., Rodriguez E., Ya-Ting S. (2013). Physical Activity and Quality of Life. J. Prev. Med. Public Health.

[44] Kupcewicz E., Grochans E., Kadučáková H., Mikla M., Jóźwik M. (2020). Analysis of the Relationship between Stress Intensity and Coping Strategy and the Quality of Life of Nursing Students in Poland, Spain and Slovakia. Int. J. Environ. Res. Public Health.

[45] Boerma T., Hosseinpoor A.R., Verdes E., Chatterji S. (2016). A global assessment of the gender gap in self-reported health with survey data from 59 countries. BMC Public Health.

[46] Grasdalsmoen M., Eriksen H.R., Lønning K.J., Sivertsen B. (2020). Physicalexercise, mental health problems, and suicide attempts in university students. BMC Psychiatry..

[47] Codina N., Pestana J.V., Valenzuela R., Giménez N. (2020). Procrastination at the Core of Physical Activity (PA) and Perceived Quality of Life: A New Approach for Counteracting Lower Levels of PA Practice. Int. J. Environ. Res. Public Health.

[48] Grzanka-Tykwińska A., Chudzińska M., Kędziora-Kornatowska K. (2014). Evaluating the quality of life of elderly people who attend classes at the University of the Third Age. Med. Biol. Sci..

[49] Czapiński J., Panek T. (2015). Social Diagnosis 2015.The objective and subjective quality of life in Poland Report. Social Monit. Council..

[50] Bouchard C., Blair S.N., Haskell W.L. (2012). Physicalactivity and Health.

[51] Konopack J.F., McAuley E. (2012). Efficacy-media-ted effects of spirituality and physical activity on quality of life: A path analysis. Health Qual. Life Outcomes.

[52] Rugbeer N., Ramklass S., Mckune A., van Heerden J. (2017). The effect of group exercise frequency on health related quality of life in institutionalized elderly. Pan Afr. Med. J. (PAMJ).

[53] Navarro-Flores E., Romero-Morales C., Becerro de Bengoa-Vallejo R., Rodríguez-Sanz D., Palomo-López P., López-López D., Losa-Iglesias M.E., Calvo-Lobo C. (2020). Sex Differences in Frail Older Adults with Foot Pain in a Spanish Population: An Observational Study. Int. J. Environ. Res. Public Healthy.

